# Pedigree analysis for the genetic management of group‐living species

**DOI:** 10.1002/ece3.1831

**Published:** 2016-04-02

**Authors:** Belén Jiménez‐Mena, Kristine Schad, Nick Hanna, Robert C. Lacy

**Affiliations:** ^1^ Bioinformatics Research CentreAarhus UniversityC.F. Møllers Allé 8DK‐8000Aarhus CDenmark; ^2^ AgroParisTechUMR1313 Génétique Animale et Biologie Intégrative16 rue Claude Bernard75231Paris 05France; ^3^ INRAUMR1313 Génétique animale et Biologie Intégrative78350Jouy‐en‐JosasFrance; ^4^ European Association of Zoos and Aquaria (EAZA)AmsterdamThe Netherlands; ^5^ Audubon Nature Institute6500 Magazine St.New OrleansLouisiana70118; ^6^ Chicago Zoological SocietyBrookfieldIllinois60513

**Keywords:** Conservation genetics, *Eurycea rathbuni*, ex situ conservation, group management, *group2PMx*, group‐living organisms, population management, Texas blind cave salamander

## Abstract

Captive breeding programs are an important tool for the conservation of endangered species. These programs are commonly managed using pedigrees containing information about the history of each individual's family, such as breeding pairs and parentage. However, there are some species that are kept in groups where it is hard to distinguish between particular individuals within the group, making it very difficult to record any information at an individual level. Currently, software and methods commonly used for registering and analyzing pedigrees to help manage populations at an individual level are not adequate for managing these group‐living species. Therefore, there is a need to further develop these tools and methodologies for pedigree analysis to better manage group‐living species. PMx is a program used for the management of ex situ populations in zoos and aquariums. We adapted the pedigree analysis method implemented in PMx to analyze pedigrees (records of descendant lineages) of group‐living species. In addition, we developed a group pedigree data entry sheet and *group2PMx,* a converter program that enables group datasets to be imported into PMx. We show how pedigree analysis of a group‐living species can be used for population management using the studbook of the endangered Texas blind cave salamander *Eurycea rathbuni*. Such analyses of the pedigree of groups can improve the management of group‐living species in ex situ breeding programs. Firstly, it enables better management decisions based on more accurate genetic measures between groups, allowing for greater control of inbreeding. Secondly, it can improve the conditions in which group‐living species are held by adapting husbandry practices to better reflect conditions of these species living in the wild. The use of the spreadsheet and *group2PMx* extends the application of PMx_,_ allowing conservation managers and other institutions outside the zoo and aquarium community to easily import and analyze their pedigree data.

## Introduction

Conservation of genetic diversity and conservation of species diversity are two of the most recognized requirements for the conservation of biodiversity (Frankham 1995). Zoos and aquariums manage ex situ breeding programs aiming to retain as much genetic diversity of these populations as possible (Ballou et al. 2010). Historically, ex situ breeding programs have contributed immensely in recovering the genetic diversity of several endangered species (Conde et al. 2011). Information about the life history of individuals (pedigrees) held in zoos and aquariums are kept in studbooks (Van Dyke 2010). The coordination and organization of ex situ breeding programs are usually based on analyzing these pedigrees to genetically manage collections and control breeding pairings. Development of methodologies for pedigree analysis has contributed substantially to the fields of animal breeding and conservation genetics, resulting in many published methods and extensive application (Flesness 1977; Ballou 1983; Foose et al. 1986; Lacy, 1989; Lacy 1994, 1995; Ballou and Lacy 1995; Lacy et al. 1995; Dunner et al. 1998; Caballero and Toro 2000; Fernández et al. 2003; Gutiérrez et al. 2009; Cervantes et al. 2011).

Pedigree analysis methods generally require complete information about the parents of every captive‐born individual. There are many species that are kept in groups for biological/husbandry needs, or for practical reasons, for example, limited institutional space (Wang 2004). For such groups, it is very difficult to register information and keep track of the life history for all individuals (Leus et al. 2011). This can be due to difficulties in (1) identifying individuals at different life stages or specifying exactly how many individuals are in a group, (2) defining in a clear way what an “individual” of a species is, and/or (3) controlling breeding pairs. Numerous species of invertebrates, amphibians, reptiles, and fish species, as well as some mammals, such as ungulates and primates, meet at least one of these criteria (Smith 2010). For such populations, applying the same management tools used at the individual level is a challenge (Leus et al. 2011). Zoos and aquariums are increasingly extending their breeding programs to help conserve endangered group‐living species (Wang 2004). It is equally important for the survival of these groups to retain their genetic diversity and monitor the rate at which they accumulate inbreeding through genetic drift, measured by the effective population size *N*_e_ (Wright 1931). A number of theoretical pedigree analysis (Wang 2004) and demographic techniques (Burlingham‐Johnson et al. 1994; Ballou et al. 2010 and Frankham et al. 2010) have been developed to manage group‐living species, so‐called *group management,* but they have not often been applied in practice (Ballou et al. 2010). Methods exist for using rotational schemes of moving individuals between groups in order to preserve genetic diversity, but these require highly structured patterns of mating (Princée 1995); they are not applicable to the irregular pedigrees that often arise when either the biology of the species or the program management results in less control over the patterns of group formation. There is a need to develop methods and practical computing tools to analyze pedigrees of group‐living species that can, in addition, deal with different breeding systems and actual population movements.

PMx (Ballou et al. 2011; Lacy et al. 2012; available at www.vortex10.org/PMx.aspx) is a program used to analyze pedigree data and perform genetic and demographic analyses. Zoo and aquarium communities manage ex situ populations using PMx with SPARKS and PopLink, two record‐keeping programs used to store pedigrees (Faust et al. 2012; ISIS, 2012). PMx can analyze pedigrees which contain uncertainty of parentage (Lacy 2012), and this can, in theory, be extended to analysis of group pedigree data (Traylor‐Holzer 2011). However, such analysis is currently limited in practice, as SPARKS and PopLink cannot handle data of group‐living species. For this reason, there are currently very few pedigrees (i.e., studbooks) kept for group‐living species and it is thus uncommon for most of these species to be genetically managed. A rare example is the Texas blind cave salamander *Eurycea rathbuni*, a cave‐dwelling amphibian from San Marcos, south‐central Texas (Hammerson and Chippindale 2004). This species is currently listed as Endangered by the state of Texas and by the U.S. Federal Government (U.S. Fish and Wildlife Service, 1967; Hammerson and Chippindale 2004). The North American AZA (Association of Zoos and Aquariums) currently manages this population (ATAG 2008), but it is not currently an official breeding program. The Texas blind cave salamander shows aggregation patterns in the wild (Epp et al. 2010), and in captivity is generally kept in groups. The identification of individuals is difficult: They do not present sexual dimorphism and are very difficult to mark or differentiate at any life stage. The application of group pedigree analysis for this species would help maintain the genetic diversity of the ex situ population, leading to a much needed successful breeding program.

In this paper, we address the challenges of group management (Leus et al. 2011) by extending pedigree analysis techniques from individual‐managed diploid species to the genetic management of group‐living diploid species. Herein, we (1) explain the terminology and genetic measures used in PMx for pedigree analysis of group‐living species, and (2) describe a new Group Management Package, which includes a user‐friendly record‐keeping template for recording pedigrees of group‐living organisms, and *group2PMx*, an executable program that converts group datasets to be readable by PMx. We provide an example of pedigree analysis of a group‐living species, the Texas blind cave salamander, and show how this can be used for the management of the population. Our aim was to provide a practical tool for the genetic management of group‐living species and encourage the use of this procedure among conservation managers and institutions involved in ex situ conservation programs.

## Methods

### Pedigree analysis of (diploid) group‐living organisms

We extend the traditional pedigree analysis of individuals to cater for different types of breeding systems associated with group management, that is, where a group is formed by a parental group reproducing (fission) or by merging different groups together (fusion). In this context, we define a group as a “gene pool”: an entity with a mixed assemblage of genes, which cannot be readily or easily divided into definable and trackable individuals. Thus, with the exception of a special case in which the numbers of males and females within a group are known (see Bisexual merge, below), we assume that nothing is known about the internal structure of each group (e.g., its sex ratio, individual variance in breeding success, nonrandom mating, or unequal probabilities of being selected during formation of progeny groups). Thus, it is assumed that new groups are formed by random sampling of alleles from the parental entities. If there were information about differential population structures – for example, different sex ratios or other breeding structures that led to different proportions of each group being successful breeders – then equations for estimating kinships could be refined to account for that additional information. However, such information would not typically be available except through identification of individual pedigrees, in which case standard pedigree analysis methods could be used to achieve greater precision in describing kinship structure and optimizing genetic management (Ballou and Lacy 1995; Lacy 1995, 2012; Ballou et al. 2010).

Note that we define the “parents” of a new group to be those one or more entities (whether groups and/or individuals) that were sampled and possibly combined to form the new group. Thus, a group's “parent” is a source of genes that went into the formation of the group, but not necessarily a genetic parent in the sense of Mendelian genetics among sexually reproducing individuals. The methods we present assume that each group remains genetically unchanged over time, as would be the case if its composition did not change as the result of births and deaths within the group. This assumption can be met if (1) generations are kept discrete, and new individuals born within the group being removed to form an offspring group in the next generation, rather than being left with the genetic parents in a mixed‐generation group, and (2) deaths or other removals of individuals from a group are recorded by designating the newly constituted subgroup as being a new group formed by sampling a set of individuals from the larger source group. The designation of a new group from the sampling of a subset of individuals does not need to be carried out with each death or removal, but rather (for the purpose of tracking the genetics within and between groups) can be carried out after the removal of multiple individuals, only at the time where genetic calculations need to be updated. If the above assumptions are not met, and there is no tracking of changes to group composition after a group is formed or a death event occurs before a birth and the population has not been updated, then the calculations below of inbreeding and kinship of a group to itself will overestimate the genetic diversity within the groups. However, the calculations of pairwise relationships of each group to other groups will remain correct, as those are not affected by the random subsampling of alleles that occurs when there are births and deaths within groups. Each allele that was present at the formation of a group has the same probability of being sampled later, because we have no information about which alleles were transmitted through even multiple episodes of subsampling.

The concept of kinship (Malécot 1948) between individuals can be directly extended to groups. The kinship coefficient *f*_*xy*_ between two groups *x* and *y* is defined as the probability that two alleles sampled from the same locus in *x* and *y* are “identical by descent” (IBD), that is, the two alleles descend from the same allele present in a common ancestor. Because within a group two alleles at a locus can come from the same parental source, the inbreeding coefficient for groups has a slightly different meaning than for individuals (for which inbreeding can be defined as the probability that the maternally derived allele is IBD to the paternally derived allele). Therefore, the inbreeding coefficient *F*_*x*_ of a group *x* refers to the probability that two randomly sampled alleles at a locus that are not the same physical allele (i.e., the sampling is without replacement) are IBD. Groups that initiated the ex situ population (and usually originated from the wild) are considered founders. As in pedigree analysis of individuals, founder groups are assumed to be nonrelated and noninbred, thus *F*_*x*_ and *f*_*xy*_ between founders are equal to 0, and *f*_*xx*_ = 1/(2*n*_*x*_), where *n*_*x*_ is the number of individuals, that is, diploid genomes, in founder group *x*. If individuals within groups are not diploid, then the methods presented here can be modified by replacing 2*n*_*x*_ in each equation with *m*_*x*_ *n*_*x*_, in which *m*_*x*_ is the number of chromosome sets in the nucleus (for diploid, *m* = 2).

A group can be formed by several different breeding systems. *F*_*x*_ and *f*_*xy*_ can be computed in different ways depending on the specific breeding system used to form group *x*. Note that the kinship of a group to itself (*f*_*xx*_) is the same for all group (and individual) breeding systems and it is given as:

(1) $$f_{xx}=\frac{1}{2n_{x}}+\left(1-\frac{1}{2n_{x}}\right)F_{x}$$

because with probability 1/(2*n*_*x*_) the same physical allele is sampled when two alleles are randomly selected from a group, and with probability (1 − 1/(2*n*_*x*_)) the sample physical allele is not resampled, in which case the probability of IBD is, by definition, the inbreeding coefficient, *F*_*x*_.

All the equations presented herein incorporate extensions for the possibility of multiple parentage and unknown ancestry, developed by Ballou and Lacy (1995) and Lacy (2012). A group can be treated similarly to an individual with multiple parents, and partially unknown ancestry due to unrecorded parents is possible with groups just as it is with individuals. To account for uncertainty in parentage, we define *G* as the set of possible parent groups *i,* and *p*_i_ the expected proportional contribution of group *i* as a parent when the new offspring group was formed. If all parents are known, then *p*_*i*_ is the proportional contribution of group *i* to the new group. If it is uncertain if *i* was a parent, then *p*_*i*_ will be the probability that *i* was a parent multiplied by the proportional contribution made to the offspring group if *i* was one of its parents. To account for any unknown ancestry, *k*_*i*_ is defined as the proportion of known (traceable) alleles contained within group *i,* that is, the portion of the alleles that come from the known ancestry in the population (Lacy 2012). For example, if a group *x* has two equally contributing parents, and one of which was of unknown origin and the other had a fully known pedigree back to the founders, then *k*_*x*_ = 0.5. In general, for a group *x* with the parental group set *G*,* k*_*x*_ is calculated iteratively as the weighted mean of the *k* values for all possible parents:

(2) $$k_{x}=\sum_{i\in G}p_{i}k_{i}$$

This methodology for considering only the portion of each group's ancestry that is traceable to founders makes the assumption that the unknown portion of each genome has the same genetic relationships to other entities as does the known portion. If instead, the unknown ancestors are assumed to be unrelated to the known portion of the population (i.e., all *k*_*i*_ values are set to 1), then kinships will be underestimated to the extent that the unknown parents were not unrelated, new founders. Moreover, including unknown portions of a group's ancestry as new genetic material for the population would have the undesirable consequence that greater genetic value would be assigned to groups that have the lowest portion of their pedigree known (Ballou and Lacy 1995).

Note that in the derivations below, *p*_*i*_ and *k*_*i*_ are always used as the product *p*_*i*_ *k*_*i*_ which represents the fraction of traceable alleles in group *x* that came from parental group *i*. At the start of the pedigree, *k*_*x*_ = 1 if *x* is wild caught (has wild parents and is assumed to be a founder) or can otherwise be assumed to be a genetically unique founder, unrelated to all other founders. If group *i* was of unknown origin and possibly related by unknown amounts to others in the pedigree, then a common practice is to exclude such unknown entities, so that the calculations of genetic metrics from the pedigree will not be biased by unwarranted assumptions of such unknown entities having no alleles IBD with other founders (Ballou and Lacy 1995; Lacy 2012). If unknown ancestries are to be excluded, then founders of unknown origin are assigned *k*_*x*_ = 0.

We cover almost every possible breeding system existing to form a group using four options: In PMx terminologies, these are MERGE, BISEXUAL MERGE, SPLIT, and EXTRACT. Specific details about these four major types of breeding systems for groups are given below. Table 1 presents a summary of the equations for the inbreeding and kinship calculations specific to each process by which a group can be formed.

**Table 1:** General equations for the group size (*n*_*x*_), proportion of the group known (*k*_*x*_), kinship (*f*_*xy*_ and *f*_*xx*_), and inbreeding coefficients (*F*_*x*_) for the different group breeding systems

Breeding system	Entity	*n*_*x*_	*k*_*x*_	*f*_*xy*_	*F*_*x*_	*f*_*xx*_
MERGE	Group	$\sum_{i\in G}c_{i}$	$\sum_{i\in G}p_{i}k_{i}$	$\frac{\sum_{i\in G}p_{i}k_{i}f_{iy}}{k_{x}}$	$\frac{\sum_{i\in G}\sum_{j\in G}p_{i}k_{i}p_{j}k_{j}f_{ij}}{k_{x}^{2}}$	$\frac{1}{2n_{x}}+\left(1-\frac{1}{2n_{x}}\right)F_{x}$
BISEXUAL MERGE	Group	$n_{s}+n_{d}=\sum_{j\in G_{s}}c_{s}+\sum_{j\in G_{d}}c_{d}$	$0.5\sum_{i\in G_{s}}k_{s}+0.5\sum_{i\in G_{d}}k_{d}$	$\frac{\sum_{i\in G}p_{i}k_{i}f_{iy}}{k_{x}}$	$\frac{\sum_{i\in G}\sum_{j\in G}p_{i}k_{i}p_{j}k_{j}f_{ij}}{k_{x}^{2}}$	$\frac{1}{2n_{x}}+\left(1-\frac{1}{2n_{x}}\right)F_{x}$
SPLIT	Group	*c*	$\sum_{i\in G}p_{i}k_{i}$	$\frac{\sum_{i\in G}p_{i}k_{i}f_{iy}}{k_{x}}$	$\frac{\sum_{i\in G}p_{i}k_{i}f_{ii}}{k_{x}}$	$\frac{1}{2n_{x}}+\left(1-\frac{1}{2n_{x}}\right)F_{x}$
EXTRACT	Individual	*1*	$\sum_{i\in G}p_{i}k_{i}$	$\frac{\sum_{i\in G}p_{i}k_{i}f_{iy}}{k_{x}}$	$\frac{\sum_{i\in G}p_{i}k_{i}f_{ii}}{k_{x}}$	0.5 + 0.5 *F*_*x*_

*c*_*i*_ = proportion of contribution from parental group *i*;* c*_*s*_  = proportion of contribution from sire group *s; c*_*d*_  = proportion of contribution from dam group *d*;* p*_*i*_  = relative contribution of parental group *i*;* k*_*x*_, *k*_*i*_, *k*_*s*_, *k*_*d*_  = mean proportion of group *x*, group *i*, sire *s* and dam *d*, respectively, that can be traced back to known founders; *f*_*xy*_  = kinship of group *x* to group *y*;* F*_*x*_  = inbreeding coefficient of group *x*;* f*_*xx*_ = kinship of group *x* to itself.

#### Merge

This term applies to a group that has been formed by combining alleles sampled from the gene pools of two or more existing groups. Merging can happen after individuals within several groups reproduce and a new group is formed by combining their offspring. Alternatively, the individuals from several groups can be combined without any prior reproduction. Because groups are treated as gene pools that are not partitioned into identifiable individuals, either method of forming a group through merging genes from prior groups amounts to a random sampling of alleles from each parental group and then merging those samples into a new single gene pool.

We obtain the proportion of a group that descends from known ancestry (*k*_*x*_) from equation (2), and then can further identify the size of the known portion of the group as *n*_*x*_ *k*_*x*_.

The kinship between groups *x* and *y* is calculated as:

(3) $$f_{xy}=f_{yx}=\frac{\sum_{i\in G}p_{i}k_{i}f_{iy}}{k_{x}}$$

as the kinship is the mean of the kinships of group y to each of the parents of group *x*, weighted by the proportional known contributions to *x*. The inbreeding coefficient is the weighted mean of all pairwise kinships between the parents that contributed to group *x*:

(4) $$F_{x}=\frac{\sum_{i\in G}\sum_{j\in G}p_{i}k_{i}p_{j}k_{j}f_{ij}}{k_{x}^{2}}$$

#### Bisexual merge

A special case of MERGE is when the sex of individuals can be identified and the total numbers of each sex counted, even though individuals are not otherwise distinguished. In this case, the parents of the new group can be partitioned into one or more sire and dam groups, *G*_*s*_ and *G*_*d*_, respectively, and we know that in the future descendants, the total genetic contribution of the sire groups must equal the total genetic contribution of the dam groups. Because this equality of the contributions of the two sexes assumes that sexual reproduction occurs, use of the bisexual merge is appropriate only when generations are kept discrete and the newly formed group will not be used as a parent for further groups before reproduction occurs within the group. In the first generation, the inbreeding coefficients for individuals within the new group are determined by the average of kinships between sires and dams. However, to be consistent with our concept of group as a mixed assemblage of genes that have been shuffled and made indistinguishable in the progeny group, inbreeding of the group will include pairs of alleles where both alleles are sampled from parental sources that are the same sex. In this case, to account for the equal contributions of male and female parents, the proportional contributions are adjusted:

$$p_{i}^{\prime }=0.5p_{i}/\sum_{i\in G_{s}}p_{i};\;\;p_{j}^{\prime }=0.5p_{j}/\sum_{j\in G_{d}}p_{j};$$

such that $\sum_{i\in G_{s}}p_{i}^{\prime }=\sum_{j\in G_{d}}p_{j}^{\prime }=0.5$. The kinship and inbreeding coefficients are then calculated as for MERGE, but using these adjusted parental contributions.

#### Split

A group is produced via SPLIT when a sample of alleles is passed on from an existing group. This may occur when members of an existing group reproduce and their offspring are partitioned into a new group. Alternatively, a new group may be formed by some individuals from an existing group. This breeding system is a special case of a MERGE, where there is only one parental group, but there may be uncertainty in which is the true parental group. The new group *x* is formed by random sampling of *n*_*x*_ individuals. In a SPLIT, the expected proportional contribution of each possible parental group, *p*_*i*_, is the probability that group *i* is the one true parent. The kinships to other groups are calculated as in equation (3), while the inbreeding coefficient is the weighted mean of the kinships of the possible parents to self:

(5) $$F_{x}=\frac{\sum_{i\in G}p_{i}k_{i}f_{ii}}{k_{x}}$$

#### Extract

This term refers to a situation where a single individual is extracted from a random group. Note that this is a special case of a SPLIT with *n*_*x*_ = 1. Although strictly speaking EXTRACT does not form a group, but rather a newly identifiable individual, we describe it here as it is commonly used in group management. Moreover, with EXTRACT being the creation of an individual from a group, and MERGE providing a means to create a groups from individuals (and/or groups), the methods presented here allow pedigree calculations and management to be conducted on any mix of individuals and groups.

The kinship of the sampled individual *x* to any other individual or group *y* and the inbreeding coefficient are calculated as with a SPLIT.

#### Optimal genetic management of groups

Strategies to manage captive breeding populations are based on the mean kinship (*MK*) value. The *MK* of an individual is defined as the average of its kinships with all individuals (including itself) (Lacy 1995; Ballou et al. 2010). Selecting breeders with the lowest MKs and, iteratively, selecting the set of breeders with the minimum mean *MK* has been shown to be the best strategy to retain genetic diversity in populations with pedigrees at individual level by both simulations (Ballou and Lacy 1995; Ivy and Lacy 2012) and empirical studies in *Drosophila* (Montgomery et al. 1997).

In the case of groups, the *MK* of group *x* can be calculated by:

(6) $$MK_{x}=\frac{\sum_{y}n_{y}k_{y}f_{xy}}{\sum_{y}n_{y}k_{y}}$$

so that the mean is weighted by the size and the proportion of known alleles of each group in the population (*k*). If group sizes are not known (e.g., if the group is formed from an uncountable number of individuals, or if changing group composition over time is not recorded as the formation of new groups from SPLITS), then each *n* in equations (6) and (7) would be set to 1, and each group would be weighted equally regardless of its (unknown) size. Such an unweighted *MK* might also be desired if the propagation of groups in the future will involve sampling equal numbers of alleles from parental groups, regardless of the group sizes. PMx provides an option to weight *MK* calculations or not by group size.

Gene diversity (*GD*) (Nei 1973) is defined as the expected heterozygosity under Hardy‒Weinberg equilibrium (Frankham et al. 2010). *GD* can be expressed as the proportion of the *GD* within the present population that has been lost relative to the source population of founders. For groups, this proportional *GD* is obtained as:

(7) $$GD=1-\frac{\sum_{x}n_{x}k_{x}MK_{x}}{\sum_{y}n_{y}k_{y}}=1-\frac{\sum_{x}\sum_{y}n_{x}k_{x}n_{y}k_{y}f_{xy}}{{\left(\sum_{y}n_{y}k_{y}\right)}^{2}}$$

#### Record‐keeping of group‐living organisms

We have developed a Group Management Package which consists of (1) a spreadsheet and (2) an executable program (*group2PMx*).

##### Excel spreadsheet template

The spreadsheet has been developed as a user‐friendly Excel file which contains the information required to analyze pedigree data for groups in PMx. This spreadsheet contains three different sections for the required information: group characteristics, parentage, and transfers between groups (moves*)*. Although the main purpose of the spreadsheet is recording of group information, it is also possible to keep the records of individuals as a substitute for the more extensive studbook records in SPARKS.

##### *group2PMx*

To import the group data into PMx from the Excel template, we have developed *group2PMx*, a Python‐based executable program. *group2PMx* converts the data from an Excel spreadsheet template into acsv file compatible with PMx.

Detailed information about the spreadsheet template and *group2PMx* can be found in the manual, which can be downloaded together with the Group Management Package from: www.vortex10.org/PMx.aspx or https://github.com/BelenJM/Group_Management.

#### An example of pedigree analysis for groups

**Figure 1:**
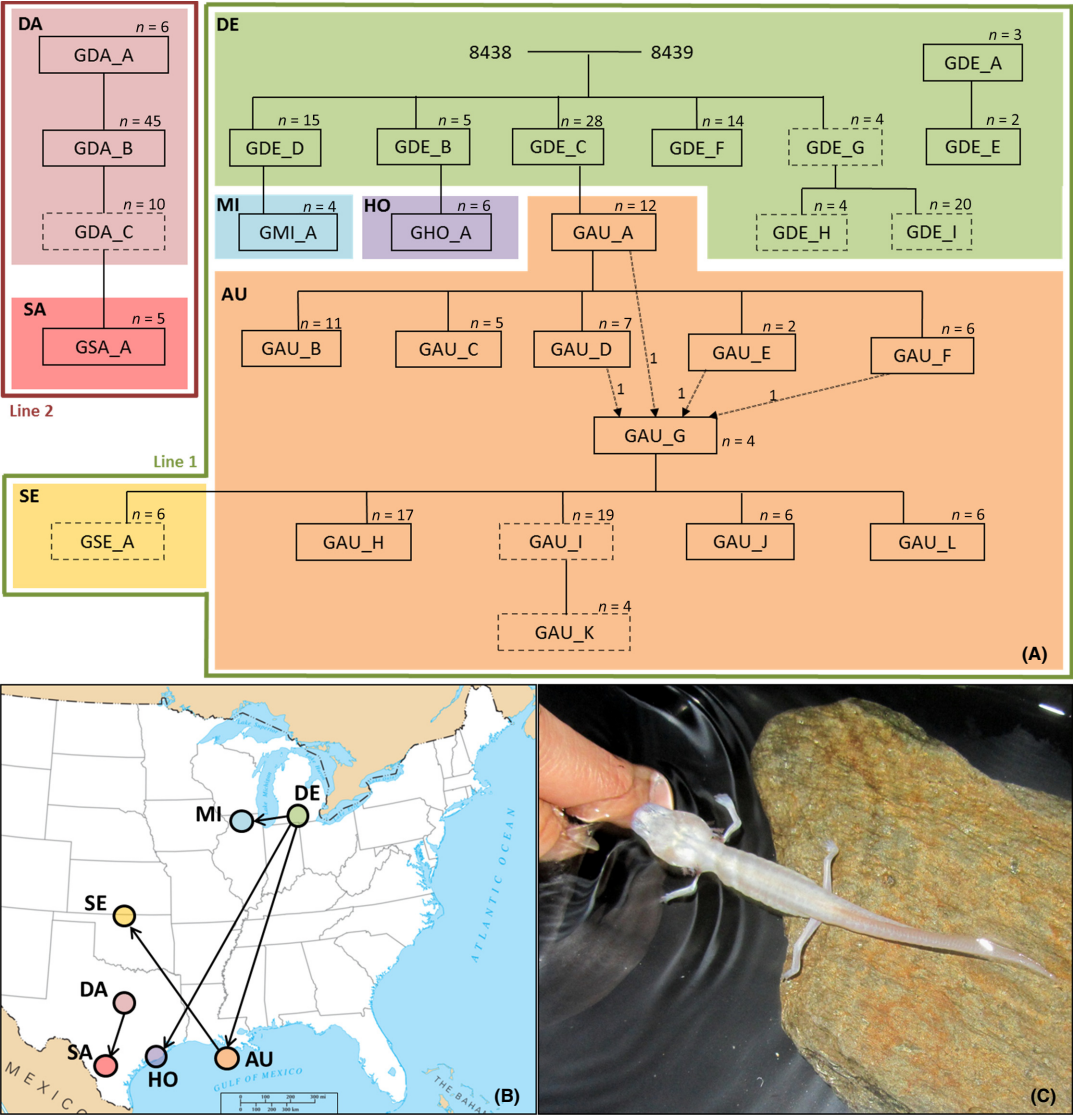
Pedigree and distribution of the ex situ population of the Texas blind cave salamander. (A) Pedigree of the ex situ population of the Texas blind cave salamander. The groups are represented as rectangles (e.g., GDE_C), and the individuals with their studbook identification number (e.g., 8438). Dashed line rectangles represent the current living groups. The size of the group (*n*) is indicated on the top‐right corner of each group. The lines connecting groups indicate a reproduction. The dashed arrows indicate a merge, in which some individuals from the original group (indicated by the number on the arrow) were transferred. Lines 1 and 2 gather the groups that descend from the same founders. The institutions are indicated using the first two letters of the city: AU (Audubon Zoo), DA (Dallas Zoo), DE (Detroit Zoological Park), SE (Sedgwick County Zoo), MI (Milwaukee), HO (Houston), and SA (San Antonio). (B) Geographical distribution of the historical and current institutions holding the ex situ population. The arrows illustrate the history of individuals' transfers between the different institutions. (C) An adult individual of the Texas blind cave salamander from the population of DE.

To demonstrate the application of pedigree analysis to group‐living organisms, the above tools and methods were applied to the studbook of the ex situ population of the Texas blind cave salamander (Appendix S1), held by seven institutions in the USA. We first translated the studbook into a group pedigree according to the group management terminology used by PMx, and stored it in the Excel spreadsheet template from the Group Management Package (Appendix S2). A visual representation of the pedigree analyzed is given in Figure 1A. The transformed pedigree spreadsheet was then converted using *group2PMx* and imported into PMx.

The previously presented genetic estimators (*f*_*xy*_, *F*_*x*_ *, MK*_*x*_ *, GD*) were calculated using PMx. The wild‐caught founders for this ex situ population were not included in the calculations of the population statistics, *MK*_*x*_ and *GD*, as recommended by Lacy (1995).

## Results

The studbook of the ex situ population of the Texas blind cave salamander contains the records of the nonliving and living populations between 1988 and 2011. To date, there have been a total of 278 individuals held in seven institutions in the USA (Audubon Zoo, AU; Dallas Zoo, DA; Detroit Zoological Park, DE; Sedgwick County Zoo, SE; Milwaukee Zoo, MI; Houston Zoological Gardens, HO; and San Antonio Zoological Gardens, SA). The current captive breeding population (Fig. 1A) consists of seven groups (a total of 67 individuals) held in four (AU, DA, DE, and SE) of the seven institutions. A map of the historical and current geographical distribution of the ex situ breeding population can be found in Figure 1B. DE has the largest number of groups (3), followed by AU (2), SE and DA (1). The current living population can be divided into two distinct lines of descent according to the shared ancestry: line 1 is formed by the groups from DE, AU, HO, and SE and was founded by two individuals from DE (IDs 8438 and 8439 in Fig. 1A), and line 2 is formed by the group of DA, founded by six individuals (GDA_A in Fig. 1A). The two founders from DE have contributed to 85% of the total living population. It is also worth noting that, unfortunately, there is no registration of the number of generations and breeding history for the group of DA, as its information was not recorded in the early stages of the breeding program. All that is known about this group is how many individuals founded it (6), the maximum past group size (45) and the current group size (10).

Table 2 shows the matrix of kinship coefficients between the living groups and between the institutions, as well as the inbreeding coefficients and the group sizes. The sizes of the groups vary between 4 and 20 individuals. The group from SE (GSE_A) and the two living groups from AU (GAU_I and GAU_K) have the largest inbreeding coefficients (0.60, 0.60, and 0.61, respectively), whereas the group from DA (GDA_C) has the lowest inbreeding coefficient (0.09). GDA_C has the lowest *MK* value (0.02); the rest of the groups present higher and very similar *MK* values, ranging from 0.34 to 0.37. GDA_C is the only group that is genetically unrelated to the groups at other institutions and its kinship value with the other groups is 0. The kinship coefficients between the rest of the groups range from 0.25 (the kinship of that of full‐siblings) to 0.62.

**Table 2:** Pairwise kinship coefficients (*f*_*xy*_ *)*, group size (*n*_*x*_), inbreeding coefficient (*F*_*x*_), and mean kinship (*MK*_*x*_) for the living groups of the ex situ breeding program of the Texas blind cave salamander

	ID	GAU_I	GAU_K	GDA_C	GDE_G	GDE_H	GDE_I	GSE_A
*f*_*xy*_	GAU_I	0.62	0.62	0	0.25	0.25	0.25	0.61
	GAU_K	0.62	0.67	0	0.25	0.25	0.25	0.61
	GDA_C	0	0	0.14	0	0	0	0
	GDE_G	0.25	0.25	0	0.56	0.56	0.56	0.25
	GDE_H	0.25	0.25	0	0.56	0.62	0.56	0.25
	GDE_I	0.25	0.25	0	0.56	0.56	0.57	0.25
	GSE_A	0.61	0.61	0	0.25	0.25	0.25	0.64
*n*_*x*_		19	4	10	4	4	20	6
*F*_*x*_		0.60	0.61	0.09	0.50	0.56	0.56	0.60
*MK*		0.37	0.37	0.02	0.34	0.35	0.35	0.37

At the institutional level (Table 3), the populations from AU, DE, and SE have high values of *MK* (>0.5), whereas DA has the lowest value (0.14). DA also has the largest *GD* (0.86), followed by DE, AU, and SE. The *GD* and *MK* of line 1 are larger and lower, respectively, than the individual values from each of the institutions that form it (AU, DE, and SE).

**Table 3:** Number of living groups (*n*_gr_), total number of individuals (*n*_ind_), size of the founder group (*n*_fo_), Gene diversity (*GD*), mean kinship (*MK*), and mean inbreeding (Mean *F*_*x*_) for the institutions that hold the living populations of the breeding program of the Texas blind cave salamander *(AU, DA, DE,* and *SE)*, for the two descent lines (lines 1 and 2) and for the total population (FULL POP, in bold). Note that *n*_fo_ often represents the same founding individuals contributing to multiple populations

Pop	*n*_gr_	*n*_ind_	*n*_fo_	*GD*	*MK*	Mean *F*_*x*_
AU	2	23	2	0.38	0.61	0.60
DA	1	10	6	0.86	0.14	0.09
DE	3	28	2	0.43	0.57	0.55
SE	1	6	2	0.36	0.63	0.60
Line 1	6	57	2	0.58	0.42	0.58
Line 2	1	10	6	0.86	0.14	0.09
FULL POP	**7**	**67**	**8**	**0.69**	**0.31**	**0.51**

AU, Audubon Zoo; DA, Dallas Zoo; DE, Detroit Zoological Park; SE, Sedgwick County Zoo.

The kinship matrices obtained from the pedigree analysis of the Texas blind cave salamander (Tables 2 and 3) reveal that GDA_C should be given priority in mating due to its very low *MK*. It is often recommended that pairings between entities with highly dissimilar *MK* be avoided, because it creates progeny that have a genome that is half valuable and half nonvaluable, making it more difficult to optimally manage the population in the future (Ballou et al. 2010). Thus, because all other groups have higher *MK*, mating between them and GDA_C should be theoretically avoided, but they are needed in practice in order to avoid accumulation of inbreeding in GDA_C, and no other possible partners for mating to group GDA_C exist. Exchanges of individuals from GDA_C population are therefore recommended for maximizing the *GD* of the overall population and maintaining the ex situ population as a metapopulation under integrated management. The remaining groups can be freely bred with groups from other institutions (e.g., GAU_I with GDE_G, but not GAU_I with GAU_K) due to their similar *MK* values. The current population of the Texas blind cave salamander was initiated from a total of eight individuals (two from DE and six from DA), with very unequal numbers of progeny. This is a low number compared to the recommendation of having a founder group of at least 20 individuals with equal contributions (Lacy 1994). The incorporation of new founder groups would decrease the *MK* values of all of the groups and increase population *GD*.

## Discussion

In this paper, we extended pedigree analysis to managing group‐living organisms and illustrated such analysis on the pedigree of the Texas blind cave salamander.

As in traditional pedigrees containing information at an individual level, ex situ breeding programs of groups would benefit from recording information about the group size at each generation of reproduction and its parental group(s). To our knowledge, there is hardly any pedigree information kept for group‐living species, due to the difficulty in recording and the lack of tools and guidelines to store datasets. Once this information about the groups has been recorded at each generation, the kinship of a group to all other groups can be estimated through the subsequent generations and optimal breeding decisions and management recommendations can be provided. Guidelines for an effective breeding program recommend that exchanges between individuals are performed according to their *MK* values (Ballou and Lacy 1995). This has been illustrated here for the management of the Texas blind cave salamander.

Breeding programs require accurate data collection and analysis (Ralls and Ballou 1986). Pedigree analysis and optimal management at an individual level is critically dependent on accurate and complete recording of the parentage for each individual. Lack of information and missing or unknown data can also negatively impact pedigree analysis of groups, which can lead to an underestimation of the kinships and inbreeding coefficients. In the case of the Texas blind cave salamander, the studbook was assembled long after the first wild individuals were captured. The DA population had been reproducing for many years before a structured breeding program was started. As a result of this incomplete history information, the genetic measures for this population may be underestimated and this can explain the large difference between the inbreeding coefficient and *MK* of GDA_C with the rest of the groups. Therefore, decisions of future transfers to other institutions based on this underestimated *MK* must be taken carefully. Nevertheless, the estimates can provide guidance to further enhance breeding management decisions, even though they might not be completely accurate.

The extension proposed in this paper can measure the loss of genetic diversity that occurs within a group's lifetime as long as groups are kept in constant sizes with discrete, nonoverlapping generations. This can be carried out if the pedigree records a SPLIT every time there is a change in the size of the group (via births, deaths or moves). The calculations in PMx would then predict the rate at which genetic diversity is lost across the generations due to the sampling that occurs at generational turnover. However, for some group‐living species it is very difficult to know if and when reproduction occurs within groups, or whether groups contain individuals from different generations. In such cases, reproduction cannot be recorded and the calculations in PMx will assume that a group remains genetically unchanged over time, is, PMx will ignore the unquantifiable loss of genetic diversity that can occur within a group's lifetime as it goes through biological generations. Even in such cases, the calculations provided by PMx might provide considerable guidance as to the relative genetic value of each group or potential group to a species management program.

Zoos and aquariums have great potential for the conservation of animal species and represent a useful tool for the genetic rescue of endangered populations (Conde et al. 2011). The tools presented in this paper can help these institutions to better manage group‐living species, such as many invertebrates, fishes, amphibians, and reptiles, that are not as well represented in zoos and aquariums as are mammals or birds (Conde et al. 2011). In addition, given that zoos and aquariums have limited resources and space to increase the number of ex situ breeding programs, the tools described here can help them to maximize the effectiveness of their breeding programs and may shift the priorities for which species are to be held in their collections. Many of these group‐living species breed quickly, have a small body size and are relatively affordable to maintain, being potentially of interest to zoos and aquariums wishing to increase the number of ex situ breeding programs in a cost–effective way (Balmford et al. 1996; Mendelson et al. 2006; Alroy 2014).

Genetic management of group‐living populations and the use of tools as presented here can have positive benefits for the long‐term health and survival of these ex situ populations. Genetic management of group‐living species, as with traditional individual management, can maintain or even reverse the loss of genetic diversity in populations, contributing to the main goal of an ex situ breeding program (Lacy 1994). Loss of genetic diversity can be very detrimental for a population (Frankham 1995) because (1) it increases homozygosity in the population causing “inbreeding depression” (Hedrick and Kalinowski 2000), leading to potential health problems and reducing the viability and fertility of the individuals, (2) the species may become more vulnerable to new or even existing diseases, and (3) in the case of a reintroduction project, limited genetic diversity may reduce the capacity to adapt to changes in the natural environment. Moreover, group management can improve the husbandry of the population managed (Smith 2010), by adjusting the way populations are kept in captivity to match the species‐specific behavior in the wild, in order to assure the success of the species development and reproduction in captivity. For instance, it has been reported that females of the Texas blind cave salamander kept in captivity only show reproductive behavior when other females are in the same area (Epp et al., 2010). Newborn snails from *Partula* spp. require direct contact with the feces of other snails to assimilate the gut flora they need for development (Burlingham‐Johnson et al. 1994). The tools described here enable the genetic monitoring of group management, making it possible to use husbandry practices that are guided by the species behavior in the wild and not constrained by traditional pedigree methods for the genetic management (Smith 2010).

The use of molecular markers is currently spreading within the field of conservation genetics as the costs of genotyping decrease rapidly (Kohn et al. 2006). However, pedigree management is more effective than molecular management in retaining genetic diversity when molecular information is used alone and the number of analyzed markers is low (Fernández et al. 2005). This is still the case for most endangered species held in zoos and aquariums, although for species with partly known or uncertain pedigrees molecular management can give greater validity to pedigree assumptions. Even though management based on group kinship is not as precise as the one based on individual kinship, it is better than no management or than treating groups as equally genetically valuable (Wang 2004). Nevertheless, it is currently unknown if *MK*‐based strategies are the most effective for managing groups (Leus et al. 2011). Recorded pedigrees for group‐living species and their analysis using the methods presented here would enable a comparison of the effectiveness of different strategies for group management. Additionally, to more directly understand how effective group management strategies are, these can be compared with the traditional management of individuals by applying group strategy to pedigrees recorded at the individual level (Leus et al. 2011).

The group management methods described here can be further improved. There are many different types of groups‐living organisms (Smith 2010), with many different reproductive strategies. Further efforts should move toward extending the pedigree analysis to deal with additional different types of group breeding systems. In addition, the assumption that each group remains genetically unchanged over time could be revisited in a further stage to account for those species in which the group genetic composition is constantly changing. The tools presented in this paper will be continually updated and improved to adapt to the needs of conservation managers. PMx is a free program that enables pedigree analysis and breeding management, but it was designed initially for use with pedigree records maintained in SPARKS and PopLink; pedigrees imported from other record‐keeping systems often need specific transformation and reformatting for use in PMx. This is generally the case when the users are from outside the zoo and aquarium community (Jansson et al. 2013). The developed Excel spreadsheet and converter make it easier to import pedigree data into the program, extending the use of PMx to management of ex situ wild populations from nonzoo and aquarium communities. Specifically, group‐living species managers and, more generally, the whole conservation community, can benefit from these tools.

## Data Accessibility

- Studbook of the Texas blind cave salamander: uploaded as online supporting information.
- Pedigree of the Texas blind cave salamander with group terminology for PMx: uploaded as online supporting information.
- Group management Package (group2PMx, Excel group pedigree template and Manual): www.vortex10.org/PMx.aspx or https://github.com/BelenJM/Group_Management.

## Conflict of Interest

None declared.

## Supporting information


**Appendix S1.** Studbook of the Texas blind cave salamander.

**Appendix S2.** Pedigree of the Texas blind cave salamander with group terminology for PMx.
