# Supplementary material for: Pedigree analysis for the genetic management of group‐living species
Source: Ecol Evol. 2016 Apr 2;6(10):3067–78. doi: 10.1002/ece3.1831 (PMC4821840; doi:10.1002/ece3.1831)
Supplement: Supplementary file 1 — Appendix S1. Studbook of the Texas blind cave salamander. [file ECE3-6-3067-s001.docx]

**Texas Blind Salamander**

*Eurycea rathbuni*

**NORTH AMERICAN REGIONAL STUDBOOK – 1^st^ Edition**


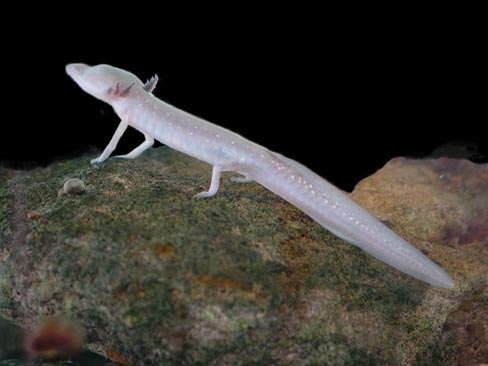


Photo courtesy Joe N. Fries, U.S. Fish and Wildlife Service

[
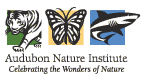
](http://audubonintranet.com/wordpress/)**Prepared by:**

Nick Hanna

Audubon Zoo

6500 Magazine Street

New Orleans, LA 70118

Phone: 504.862.0159

Fax: 504.212.5241

Email: [nhanna@auduboninstitute.org](mailto:nhanna@auduboninstitute.org)

Current to 6/20/2011

**TABLE OF CONTENTS**

**DISCLAIMER……………………………………………………………….….3**

**EXPLANATION OF DATA & CONVENTIONS………………………….…4**

**LIVING POPULATION…………………………………………………..…....5**

**HISTORICAL POPULATION……...…………………….……………….…..6**

**MNEMONICS FOR CURRENT INSTITUTIONS...……..………..….……..9**

**LINKS TO NATURAL HISTORY AND CONSERVATION INFO……….10**

**ACKNOWLEDGEMENTS**

I would like to thank Audubon Zoo for their support and the Herpetology staff; David Heckard, Melanie Litton, Kim Boyer, and Jessica Jones.

Also thanks to all the registrars, curators, and keepers and their respective institutions for providing the information necessary to complete this studbook.

### **AZA Regional Studbook Disclaimer**

Copyright 2012 by Audubon Nature Institute. All rights reserved. No part of this publication may be reproduced in hard copy, machine-readable or other forms without advance written permission from the Audubon Nature Institute. Members of the Association of Zoos and Aquariums (AZA) may copy this information for their own use as needed.

The information contained in this studbook has been obtained from numerous sources believed to be reliable. AZA and the Audubon Nature Institute make a diligent effort to provide a complete and accurate representation of the data in its reports, publications, and services. However, AZA and the Audubon Nature Institute do not guarantee the accuracy, adequacy, or completeness of any information. AZA and the Audubon Nature Institute make no warranties or representations of any kind, express or implied, including but not limited to warranties of merchantability of fitness for particular purpose. AZA and the Audubon Nature Institute disclaim all liability for errors or omissions that may exist and shall not be liable for any incidental, consequential, or other damages (whether resulting from negligence or otherwise) including, without limitation, exemplary damages or lost profits arising out of or in connection with the use of this publication.

Because the technical information provided in the studbook can easily be misread or misinterpreted unless properly analyzed, AZA and Audubon Nature Institute strongly recommend that users of this information consult with the Studbook Keeper in all matters related to data analysis and interpretation.

**EXPLANATION OF DATA & CONVENTIONS**

The data for this population has been modified from the traditional way studbooks are kept. It is not possible or practical to track these animals on an individual level, or easily sex them (although when females are gravid, eggs can easily be seen through the translucent skin). Due to the current management style and life history, tracking events such as who reproduced within a group setting is almost impossible. This creates records with limited information and the data does not work well with the conventional ways of maintaining a studbook (SPARKS or PopLink). Some specimens were given individual local ID’s, while most were and are tracked as groups. This studbook is an attempt to take all of the data, both for individuals and groups, and pull out the relevant information and standardize it. No studbook numbers have been assigned at this time, and the population will be managed by strains (“Detroit”, “Dallas”, etc.). Currently there is an effort to obtain more founders. If and when this occurs, the genetic management of this population will be revisited.

As an example and explanation of one institution’s data, the Dallas Zoo group was brought in to the institution in 1993. The group was presumably wild caught stock acquired from Southwest Texas State University. This group of animals has been maintained and reproduced for several generations under one local ID. The group started out as 6 animals, was as high as 45 animals and at the time this studbook was published, now resides at 10.

Clutches usually hatch over the course of several days. For group records, the last recorded hatch for the group was used as the group hatch date in this studbook. Similarly, the last recorded death for a group was used as the death date.

The Source/Parentage data field indicates either where the individual(s) were acquired from or what institution they hatched at and the corresponding parent group (or individuals).

**LIVING POPULATION**

| **Institution** | **Local ID** | **# of Individuals** | **Hatch/Acquisition** | **Source/Parentage** | **Last Updated** |
| --- | --- | --- | --- | --- | --- |
| Audubon | 102754 (Group) | 0.0.19 | 2/27/2009 | Audubon/100874 & 102780 | 12/1/2011 |
| Audubon | 103338 (Group) | 0.0.4 | 4/28/2011 | Audubon/102754 | 12/1/2011 |

| Dallas | 00A619 (Group) | 0.0.10 | 4/19/1993 | Dallas/00A619 | 6/20/2011 |
| --- | --- | --- | --- | --- | --- |

| Detroit | G425 (Group) | 0.0.4 | 3/25/2006 | Detroit/8438 & 8439 | 6/20/2011 |
| --- | --- | --- | --- | --- | --- |
| Detroit | G471 (Group) | 0.0.4 | 3/20/2008 | Detroit/G425 | 6/20/2011 |
| Detroit | G488 (Group) | 0.0.20 | 6/23/2009 | Detroit/G425 | 6/20/2011 |

| Sedgwick | TXBSAL (Group) | 0.0.6 | 3/15/2010 | Audubon/102780 | 1/10/2012 |
| --- | --- | --- | --- | --- | --- |

**HISTORICAL POPULATION**

| **Institution** | **Local ID** | **# of Individuals** | **Hatch/Acquisition** | **Death/Disposition** | **Source/Parentage** |
| --- | --- | --- | --- | --- | --- |
| Audubon | 100864 | 0.0.1 | 12/12/2001 | 5/8/2008 | Detroit/G271 |
| Audubon | 100865 | 0.0.1 | 12/12/2001 | 5/8/2008 | Detroit/G271 |
| Audubon | 100868 | 0.0.1 | 12/12/2001 | 10/6/2008 | Detroit/G271 |
| Audubon | 100867 | 0.0.1 | 12/12/2001 | 10/6/2008 | Detroit/G271 |
| Audubon | 100868 | 0.0.1 | 12/12/2001 | 12/31/2007 | Detroit/G271 |
| Audubon | 100869 | 0.0.1 | 12/12/2001 | 1/22/2008 | Detroit/G271 |
| Audubon | 100870 | 0.0.1 | 12/12/2001 | 1/22/2008 | Detroit/G271 |
| Audubon | 100871 | 0.0.1 | 12/12/2001 | 3/5/2008 | Detroit/G271 |
| Audubon | 100872 | 0.0.1 | 12/12/2001 | 9/6/2008 | Detroit/G271 |
| Audubon | 100873 | 0.0.1 | 12/12/2001 | 9/6/2008 | Detroit/G271 |
| Audubon | 100874 | 0.0.1 | 12/12/2001 | 9/9/2009 | Detroit/G271 |
| Audubon | 100875 | 0.0.1 | 12/12/2001 | 4/24/2007 | Detroit/G271 |
| Audubon | 101280 | 0.0.1 | 2/6/2004 | 7/23/2006 | Audubon/MULT1 |
| Audubon | 101299 | 0.0.1 | 6/26/2004 | 11/10/2004 | Audubon/MULT1 |
| Audubon | 101300 | 0.0.1 | 6/29/2004 | 11/10/2004 | Audubon/MULT1 |
| Audubon | 101396 | 0.0.1 | 6/28/2004 | 4/18/2010 | Audubon/MULT1 |
| Audubon | 101397 | 0.0.1 | 8/8/2004 | 11/10/2004 | Audubon/MULT1 |
| Audubon | 101411 | 0.0.1 | 8/24/2004 | 8/30/2004 | Audubon/MULT1 |
| Audubon | 101412 | 0.0.1 | 8/24/2004 | 9/1/2004 | Audubon/MULT1 |
| Audubon | 101413 | 0.0.1 | 8/24/2004 | 11/10/2004 | Audubon/MULT1 |
| Audubon | 101414 | 0.0.1 | 8/24/2004 | 11/10/2004 | Audubon/MULT1 |
| Audubon | 101415 | 0.0.1 | 8/24/2004 | 11/10/2004 | Audubon/MULT1 |
| Audubon | 101416 | 0.0.1 | 8/24/2004 | 11/10/2004 | Audubon/MULT1 |
| Audubon | 101664 | 0.0.1 | 3/3/2005 | 5/11/2005 | Audubon/MULT1 |
| Audubon | 101665 | 0.0.1 | 3/3/2005 | 5/11/2005 | Audubon/MULT1 |
| Audubon | 101666 | 0.0.1 | 3/3/2005 | 5/11/2005 | Audubon/MULT1 |
| Audubon | 101667 | 0.0.1 | 3/3/2005 | 5/11/2005 | Audubon/MULT1 |
| Audubon | 101668 | 0.0.1 | 3/3/2005 | 5/11/2005 | Audubon/MULT1 |
| Audubon | 101778 | 0.0.1 | 7/17/2005 | 9/16/2005 | Audubon/MULT1 |
| Audubon | 101779 | 0.0.1 | 7/17/2005 | 9/16/2005 | Audubon/MULT1 |
| Audubon | 101780 | 0.0.1 | 7/17/2005 | 9/16/2005 | Audubon/MULT1 |
| Audubon | 101781 | 0.0.1 | 7/17/2005 | 9/16/2005 | Audubon/MULT1 |
| Audubon | 101782 | 0.0.1 | 7/17/2005 | 9/16/2005 | Audubon/MULT1 |
| Audubon | 101783 | 0.0.1 | 7/17/2005 | 9/16/2005 | Audubon/MULT1 |
| Audubon | 101861 | 0.0.1 | 10/15/2005 | 3/25/2009 | Audubon/MULT1 |
| Audubon | 101951 | 0.0.1 | 5/5/2006 | 7/23/2006 | Audubon/MULT1 |
| Audubon | 101995 | 0.0.1 | 7/15/2006 | 4/18/2010 | Audubon/MULT1 |
| **Institution** | **Local ID** | **# of Individuals** | **Hatch/Acquisition** | **Death/Disposition** | **Source/Parentage** |
| Audubon | 102204 (Group) | 0.0.6 | 5/9/2007 | 4/18/2010 | Audubon/MULT1 |
| Audubon | 102687 (Group) | 0..0.17 | 11/10/2008 | 4/29/2010 | Audubon/100874 & 102780 |
| Audubon | 102754 (Group) | 0.0.19 | 2/27/2009 |  | Audubon/100874 & 102780 |
| Audubon | 102778 (Group) | 0.0.6 | 4/14/2009 | 12/9/2009 | Audubon/100874 & 102780 |
| Audubon | 102780 (Group) | 0.0.4 |  | 4/18/2010 |  |
| Audubon | 103338 (Group) | 0.0.4 | 4/28/2011 |  | Audubon/102754 |
| Audubon | 103475 (Group) | 0.0.6 | 3/15/2010 |  | Audubon/102780 |

| Dallas | 00A619 (Group) | 0.0.10 | 4/19/1993 |  | SWTEXSTUN/UNKNOWN |
| --- | --- | --- | --- | --- | --- |

| Detroit | 8438 | 0.0.1 | 10/5/2000 | 11/19/2006 | USFWS/WILD |
| --- | --- | --- | --- | --- | --- |
| Detroit | 8439 | 0.0.1 | 10/5/2000 | 4/8/2007 | USFWS/WILD |
| Detroit | 8440 | 0.0.1 | 10/5/2000 | 9/26/2003 | USFWS/WILD |
| Detroit | 8441 | 0.0.1 | 10/5/2000 | 9/27/2003 | USFWS/WILD |
| Detroit | 8442 | 0.0.1 | 10/5/2000 | 9/29/2003 | USFWS/WILD |
| Detroit | G270 (Group) | 0.0.5 | 11/18/2001 | 12/12/2001 | Detroit/8438 & 8439 |
| Detroit | G271 (Group) | 0.0.28 | 1/9/2002 | 1/5/2003 | Detroit/8438 & 8439 |
| Detroit | G309 (Group) | 0.0.15 | 8/3/2002 | 10/1/2003 | Detroit/8438 & 8439 |
| Detroit | G335 (Group) | 0.0.2 | 7/5/2003 | 10/2/2003 | Detroit/8440 & 8441 & 8442 |
| Detroit | G416 (Group) | 0.0.14 | 11/12/2005 | 2/13/2006 | Detroit/8438 & 8439 |
| Detroit | G425 (Group) | 0.0.4 | 3/25/2006 |  | Detroit/8438 & 8439 |
| Detroit | G471 (Group) | 0.0.4 | 3/20/2008 |  | Detroit/G425 |
| Detroit | G488 (Group) | 0.0.20 | 6/23/2009 |  | Detroit/G425 |

| Houston | G20444 (Group) | 0.0.6 | 5/1/2003 | 8/19/2005 | Detroit/G270 |
| --- | --- | --- | --- | --- | --- |

| Milwaukee | H1195 | 0.0.1 | 10/19/2000 | 12/27/2001 | Dallas/00A619 |
| --- | --- | --- | --- | --- | --- |
| Milwaukee | H1196 | 0.0.1 | 10/19/2000 | 12/27/2001 | Dallas/00A619 |
| Milwaukee | H1197 | 0.0.1 | 10/19/2000 | 8/3/2001 | Dallas/00A619 |
| Milwaukee | H1198 | 0.0.1 | 10/19/2000 | 4/10/2004 | Dallas/00A619 |
| Milwaukee | H1359 (Group) | 0.0.4 | 10/1/2003 | 10/20/2003 | Detroit/G309 |

| **Institution** | **Local ID** | **# of Individuals** | **Hatch/Acquisition** | **Death/Disposition** | **Source/Parentage** |
| --- | --- | --- | --- | --- | --- |
| San Antonio | 881436 | 0.0.1 | 4/28/1988 | 5/10/1988 | LONGLEY G/UNKNOWN |
| San Antonio | 890768 | 0.0.1 | 7/14/1989 | 10/23/1989 | LONGLEY G/UNKNOWN |
| San Antonio | O00050 | 0.0.1 | 10/19/2000 | 1/28/2005 | Dallas/00A619 |
| San Antonio | O00051 | 0.0.1 | 10/19/2000 | 6/14/2007 | Dallas/00A619 |
| San Antonio | O00052 | 0.0.1 | 10/19/2000 | 3/18/2004 | Dallas/00A619 |
| San Antonio | O00053 | 0.0.1 | 10/19/2000 | 12/11/2003 | Dallas/00A619 |
| San Antonio | O00054 | 0.0.1 | 10/19/2000 | 3/28/2003 | Dallas/00A619 |

**MNEMONICS FOR CURRENT INSTITUTIONS**

| **Mnemonic** | **Institution** |
| --- | --- |
| Audubon | Audubon Zoo, New Orleans, LA, USA |
| Dallas | Dallas Zoo, Dallas, TX, USA |
| Detroit | Detroit Zoological Park, Royal Oak, MI, USA |
| Sedgwick | Sedgwick County Zoo, Wichita, KS, USA |

**LINKS TO NATURAL HISTORY & CONSERVATION INFORMATION**

[**http://amphibiaweb.org/cgi/amphib_query?where-genus=Eurycea&where-species=rathbuni**](http://amphibiaweb.org/cgi/amphib_query?where-genus=Eurycea&where-species=rathbuni)

[**http://www.iucnredlist.org/apps/redlist/details/39262/0**](http://www.iucnredlist.org/apps/redlist/details/39262/0)

[**http://www.fws.gov/fisheries/nfhs/BlindS.htm**](http://www.fws.gov/fisheries/nfhs/BlindS.htm)

[**http://www.tpwd.state.tx.us/huntwild/wild/species/blindsal/**](http://www.tpwd.state.tx.us/huntwild/wild/species/blindsal/)
